# Case Report: Leiomyosarcoma of the inferior vena cava presenting as a right atrial mass: the value of multimodality imaging

**DOI:** 10.3389/fcvm.2026.1910458

**Published:** 2026-08-28

**Authors:** Ann-Sophie Eggers, Jelena Jovanić, Stefan Pahl, Cyrill Meuwly, Bojana Ilić, Christoph Philipp Kemna, Patrick Doeblin, Stefanie Maria Werhahn, Ingo Hilgendorf, Sebastian Kelle

**Affiliations:** 1 Department of Cardiology, Angiology and Intensive Care Medicine, Deutsches Herzzentrum der Charité – Medical Heart Center of Charité and German Heart Institute Berlin, Campus Virchow-Klinikum, Berlin, Germany; 2 Charité – Universitätsmedizin Berlin, Corporate Member of Freie Universität Berlin and Humboldt-Universität zu Berlin, Berlin, Germany; 3 German Centre for Cardiovascular Research (DZHK), Partner Site Berlin, Berlin, Germany; 4 Institute of Pathology, Campus Mitte, Berlin Institute of Health, Berlin, Germany; 5 Department of Cardiology, University Clinical Centre of Republic of Srpska, Banja Luka, Bosnia and Herzegovina; 6 Faculty of Medicine, University of Banja Luka, Republic of Srpska, Bosnia and Herzegovina; 7 Centre for Cardiovascular Computed Tomography and Cardiac Magnetic Resonance Imaging, Institute for Cardiovascular Diseases Dedinje, Belgrade, Serbia; 8 Department of Hematology, Oncology, and Cancer Immunology, Charité – Universitätsmedizin Berlin, Corporate Member of Freie Universität Berlin, Humboldt-Universität zu Berlin, and Berlin Institute of Health, Berlin, Germany

**Keywords:** cardiac tumor, inferior vena cava, leiomyosarcoma, multimodality imaging, right atrium

## Abstract

Leiomyosarcoma of the inferior vena cava (IVC) is a rare malignant tumor that may extend into the right atrium and mimic a primary cardiac neoplasm. Because clinical presentation is often non-specific and depends on tumor location and extent, diagnosis may be delayed, particularly in acute settings with suboptimal imaging conditions. We report the case of a 33-year-old woman presenting with progressive dyspnea, ascites and peripheral edema consistent with right-sided heart failure. Initial contrast-enhanced computed tomography, performed to exclude pulmonary embolism, suggested a right atrial myxoma. However, stepwise multimodality imaging including transthoracic and transesophageal echocardiography, multiphasic computed tomography (CT), and cardiac magnetic resonance imaging (CMR) revealed a large intravascular mass originating from the IVC with contiguous extension in the right atrium, causing near-complete luminal obstruction of atrium and IVC. CMR was essential in establishing the diagnosis by demonstrating tumor continuity, tissue heterogeneity, and malignant enhancement patterns. Histopathological analysis confirmed a low-grade spindle cell leiomyosarcoma (G2, Ki-67 approximately 40%). Staging with multiphasic CT and magnetic resonance imaging of the brain excluded distant metastases. The patient was initiated on neoadjuvant chemotherapy with doxorubicin and ifosfamide with planned surgical resection. This case illustrates the importance of multimodality imaging, with CMR playing a central role, in distinguishing malignant intravascular tumors from benign intracardiac masses. Early diagnosis and a multidisciplinary approach are essential for optimal management.

## 1 Introduction

Leiomyosarcoma of the inferior vena cava (IVC) is a rare soft tissue sarcoma accounting for approximately 5% of all leiomyosarcomas (1). However, it is the most common primary malignancy of the IVC and arises from smooth muscle cells of the venous wall with intraluminal, extraluminal, or mixed growth patterns (2). Due to its slow growth and non-specific symptoms, diagnosis is often delayed. Clinical manifestations depend on tumor location and the degree of venous obstruction. In rare cases, tumor extension into the right atrium may occur, leading to right-sided heart failure and significant hemodynamic compromise (3). We present a rare case of locally advanced intravascular leiomyosarcoma of the IVC extending into the right atrium, initially suspected to be an atrial myxoma, highlighting the diagnostic value of multimodality imaging.

## 2 Case presentation

A 33-year-old woman presented to her general practitioner with progressive dyspnea, abdominal distension, and bilateral lower extremity edema. Her past medical history was unremarkable apart from endometriosis, with no known allergies or previous relevant infections. Dyspnea had first been noted on exertion during the summer months preceding admission. In late December 2025, the patient experienced an acute worsening of dyspnea at rest, accompanied by new-onset chest pain, which she described as sharp and occurring both at rest and on exertion, and a dull, pressure-like discomfort in the upper abdomen with a sensation of tightness. Over the four weeks before admission her exercise tolerance declined further, and she was ultimately limited to climbing a single flight of stairs without pausing. Bilateral lower leg edema had developed in the week before admission, with further abdominal distension over the preceding days. She also reported nausea and loss of appetite without vomiting. She was referred to an outpatient cardiologist, where ascites and bilateral leg edema were noted, and transthoracic echocardiography identified a right atrial mass of approximately 3 cm × 3 cm. Diuretic therapy with torasemide was started, with little improvement, and she was referred to our emergency department with suspected atrial myxoma for further evaluation and inpatient workup.

**Figure 1:**
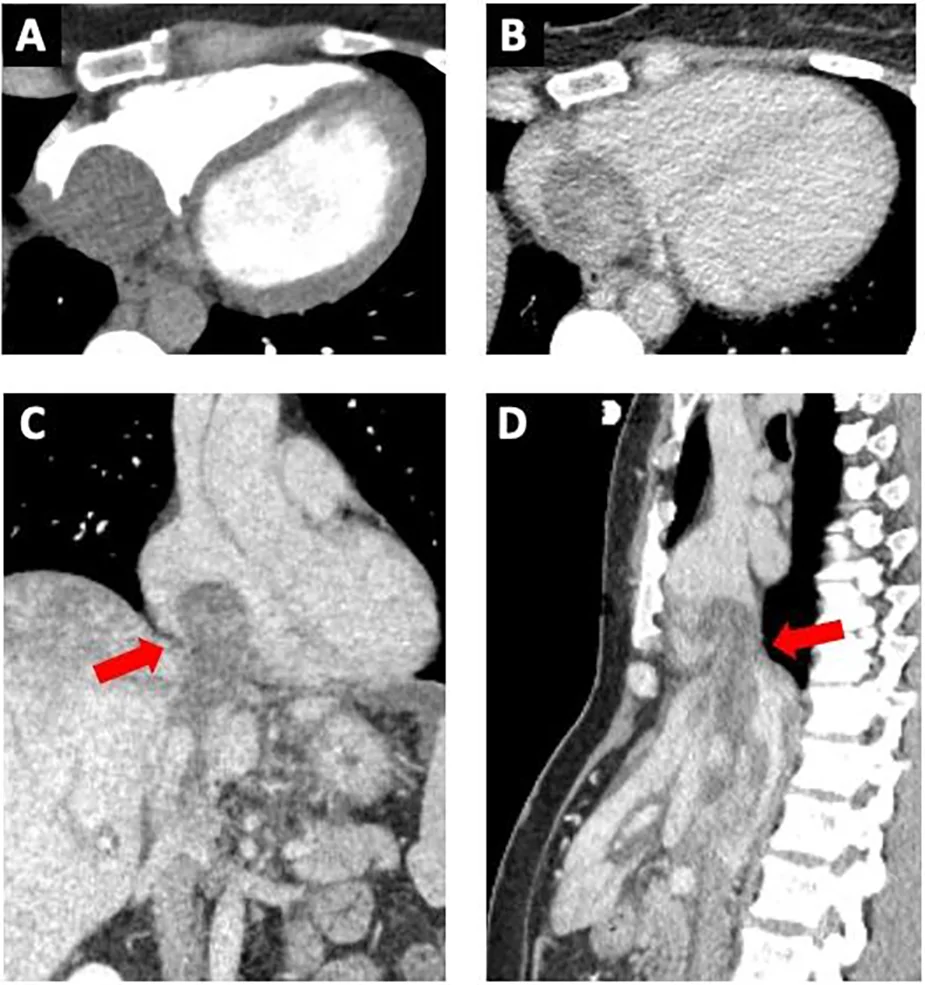
Contrast-enhanced computed tomography from different acquisitions demonstrating an inferior vena cava tumor with extension into the right atrium. **(A)** Pulmonary arterial phase in axial view, performed for suspected pulmonary embolism, showing a filling defect in the right atrium and initially suggesting a primary cardiac mass. **(B)** Axial view in venous phase demonstrating the tumor within the right atrium. **(C)** Coronal reconstruction in venous phase showing continuity of the tumor from the inferior vena cava into the right atrium (arrow). **(D)** Sagittal reconstruction confirming continuity of the tumor from the inferior vena cava into the right atrium (arrow), without evidence of regional metastases.

**Figure 2:**
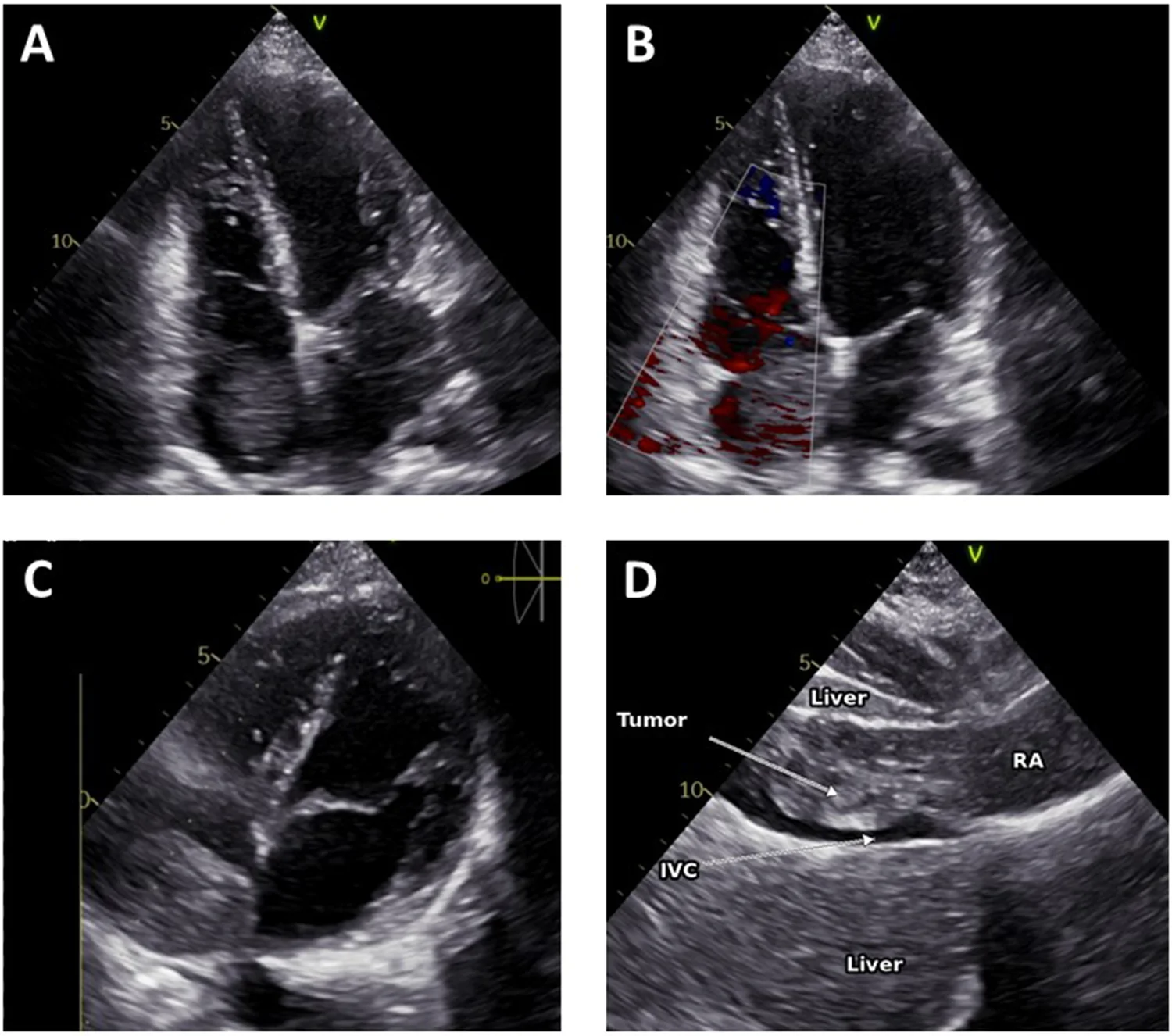
Transthoracic and transesophageal echocardiography demonstrating a right atrial mass. **(A)** Transthoracic echocardiography in four-chamber view showing a mass in the right atrium. **(B)** Color Doppler imaging demonstrating internal vascularity. **(C)** Transesophageal echocardiography showing detailed visualization of the tumor in the right atrium. **(D)** Subcostal view showing the mass at the junction between the inferior vena cava and the right atrium. IVC, inferior vena cava; RA, right atrium.

On admission, the patient was alert, fully oriented, hemodynamically stable, and afebrile, with a heart rate of 118 bpm, blood pressure of 132/95 mmHg, oxygen saturation of 96% on room air, and an ECOG performance status of 1. Her body weight was 79 kg, approximately 9 kg above her reported baseline weight of 70 kg. Cardiac auscultation revealed regular heart sounds without pathological murmurs. Lung auscultation demonstrated vesicular breath sounds bilaterally without added sounds. Abdominal examination revealed a distended but soft, non-tender abdomen with normal bowel sounds. Abdominal ultrasound showed ascites, estimated at approximately 500 mL and distributed perihepatic and in the bilateral lower abdomen, not warranting paracentesis at that time. Bilateral pitting edema of the lower extremities extending to the lower legs was present, with recoil within a few seconds. No peripheral lymphadenopathy was detected. Together with these findings, the weight gain was consistent with fluid retention. The initial laboratory report showed elevated C-reactive protein of 49.8 mg/L, creatinine of 1.03 mg/dL (reference range 0.5–0.9 mg/dL; no prior baseline available) and a normal NT-proBNP of 85 ng/L. Due to elevated D-dimer of 4.23 mg/L contrast-enhanced computed tomography (CT) of the chest was performed to exclude pulmonary embolism (Figure 1A). Even though pulmonary embolism was ruled out, a large mass in the right atrium, measuring 3.9 cm × 3.5 cm was revealed, again raising the suspicion of atrial myxoma. Transthoracic and transesophageal echocardiography confirmed a large, echodense, minimally mobile, vascularized mass adjacent to the orifice of the inferior vena cava within the right atrium, extending into the IVC and causing high-grade luminal obstruction. The IVC itself was markedly dilated, measuring 3 cm in diameter. The right ventricle was not dilated, with a basal diameter below 41 mm, and systolic function was preserved, with a TAPSE of 19 mm. Left ventricular ejection fraction was preserved. No evidence of elevated systolic pulmonary artery pressure was found. The tricuspid valve was morphologically normal, without stenosis and with only minimal regurgitation, and showed no connection to the mass. No stalk or attachment to the interatrial septum was identified (Figure 2). On the basis of these findings, myxoma and an intravascular tumor were considered the leading differential diagnoses, whereas thrombus appeared less likely given the internal vascularity of the mass.

**Figure 3:**
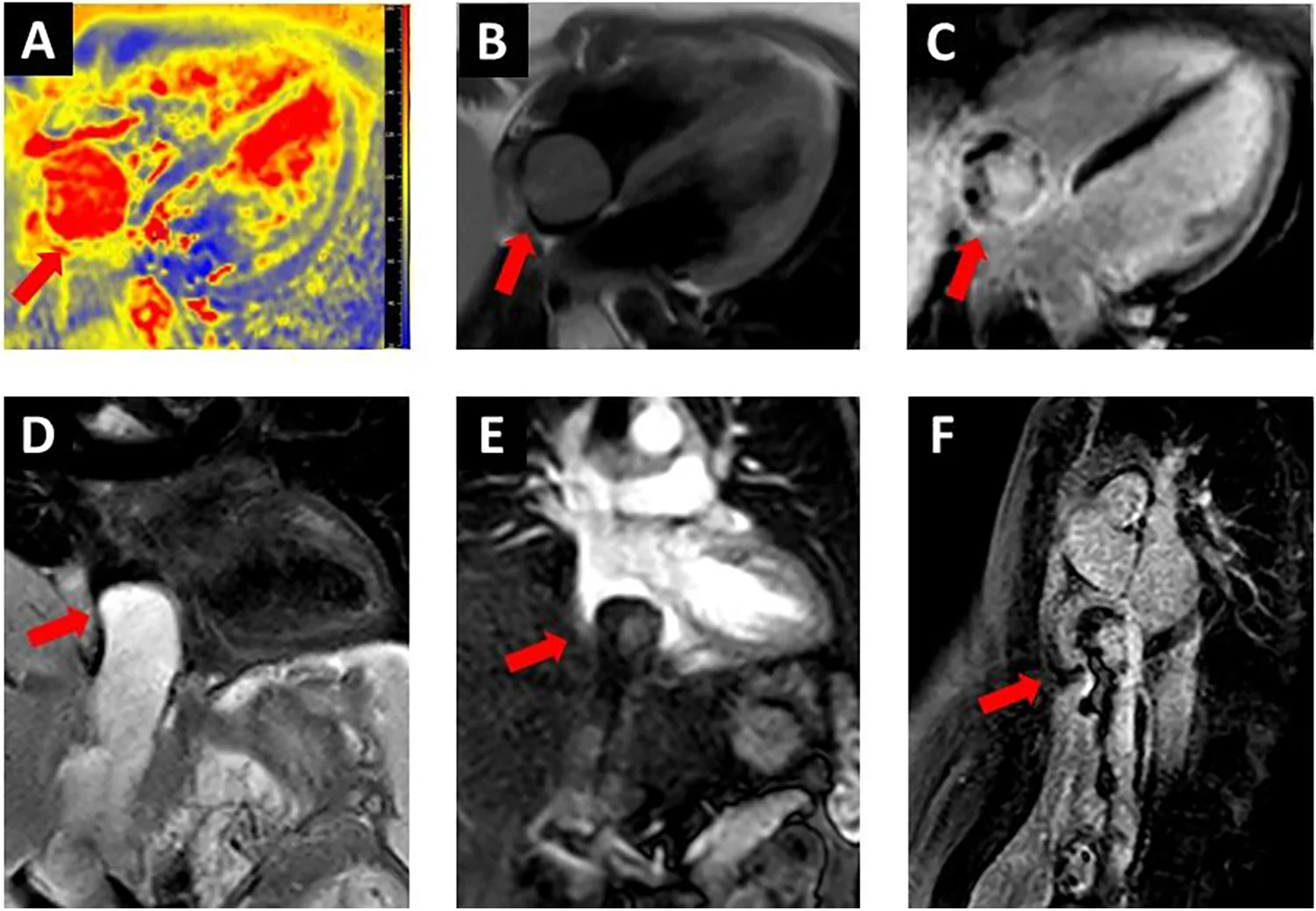
Cardiac magnetic resonance imaging enabling tissue characterization and identification of tumor origin. **(A)** T2 mapping illustrating increased T2-relaxation time. **(B)** T1-weighted black-blood four-chamber view showing a mass in the right atrium. **(C)** Late gadolinium enhancement in axial view demonstrating heterogeneous enhancement with non-enhancing areas suggestive of necrosis. **(D)** T2-weighted imaging in coronal view showing hyperintensity of the tumor. **(E)** First-pass perfusion imaging demonstrating slightly delayed enhancement compared to the left ventricular myocardium, suggesting non-coronary blood supply. **(F)** Sagittal late gadolinium enhancement confirming continuity between the inferior vena cava and the right atrium.

For further diagnostic, cardiac magnetic resonance imaging (CMR) was performed, revealing an intravascular mass extending from the IVC into the right atrium with near-complete filling of the IVC as well as right atrium and thrombotic deposits adherent to the tumor surface (Figure 3). Moreover, thrombotic material in hepatic veins and portal branches was found. As part of staging, multiphasic CT demonstrated no involvement of thoracic or abdominal lymph nodes and magnetic resonance imaging (MRI) of the brain showed no evidence of distant metastases.

**Figure 4:**
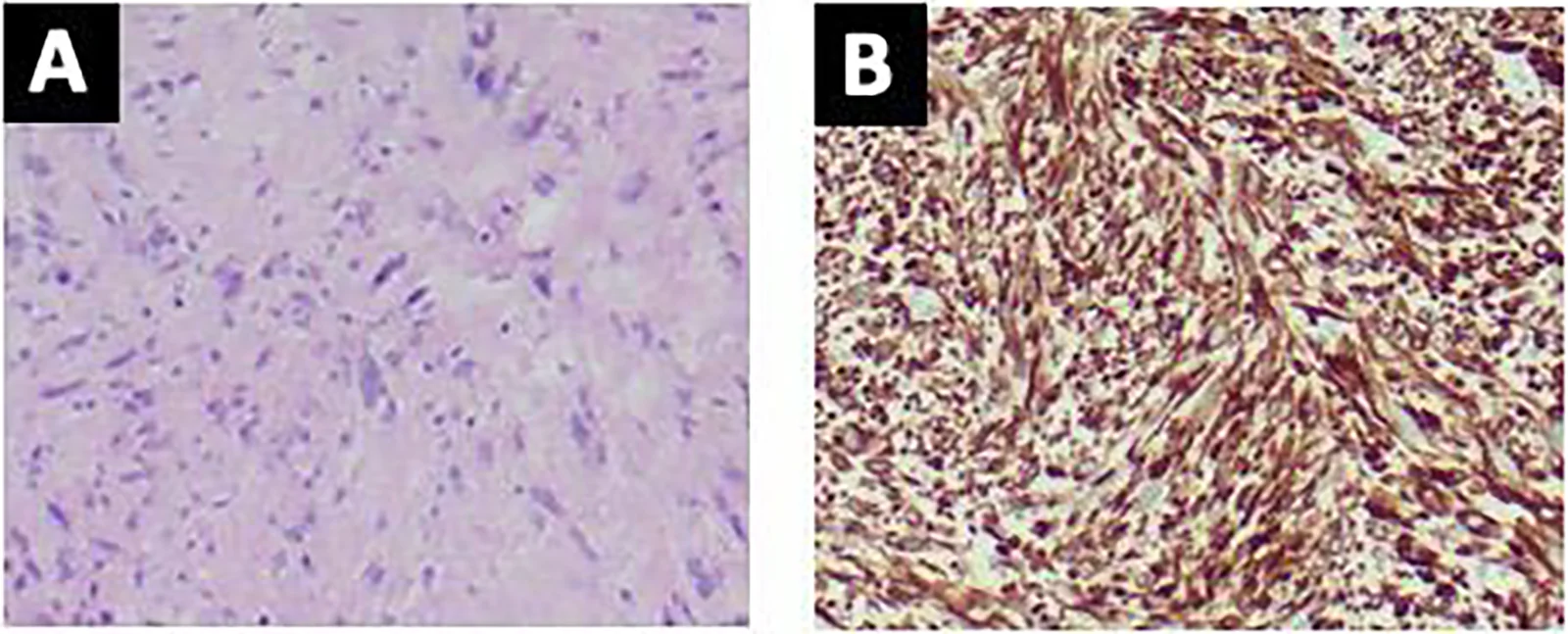
**(A)** Hematoxylin and eosin staining at ×40 magnification showing a fasciculated spindle cell neoplasm with characteristic cigar-shaped nuclei. **(B)** Immunohistochemistry demonstrated positive staining for desmin at ×100 magnification.

Subsequently, a biopsy was performed under CT guidance. Histopathological analysis of the biopsy specimen showed a fasciculated spindle cell neoplasm with cigar-shaped nuclei consistent with leiomyosarcoma (grade G2). Immunohistochemistry demonstrated positive staining for desmin and smooth muscle actin, indicating smooth muscle differentiation, with vimentin positivity confirming the mesenchymal nature of the tumor. Staining for S100, CD31, CD34, and pancytokeratins was negative, arguing against neural, vascular, epithelial, and other spindle cell neoplasms. MDM2 expression was also negative, arguing against dedifferentiated liposarcoma. The Ki-67 proliferation index was approximately 40% (Figure 4). The patient was started on neoadjuvant chemotherapy with doxorubicin and ifosfamide, in line with guideline recommendations for high-risk soft tissue sarcoma, with the aim of reducing tumor burden before surgical resection. Therapeutic anticoagulation was initiated with nadroparin 0.8 mL twice daily because of the extensive tumor-associated thrombosis, with anti-Xa levels monitored to guide dosing. Decongestion was managed cautiously, initially with intravenous furosemide 20 mg twice daily, subsequently converted to oral torasemide 5 mg once daily as fluid balance improved. The patient tolerated the first two cycles of chemotherapy well, with only mild adverse effects, including nausea, fatigue and diarrhea. Clinical signs of right-sided heart failure improved under diuretic therapy, with serum creatinine normalizing to 0.6 mg/dL, consistent with prerenal congestion at admission rather than intrinsic renal impairment. At discharge, the patient was hemodynamically stable, afebrile, and showed no evidence of peripheral edema. For further follow-up, ongoing outpatient oncological follow-up was arranged. Prior to the fourth cycle of chemotherapy, staging CT and TTE are planned to evaluate treatment response and to determine the optimal timing of surgical resection, which is currently planned following completion of 3–6 cycles of chemotherapy (Table 1).

**Table 1:** Timeline of clinical events, diagnostic workup, and treatment.

Timepoint	Event
5 months before admission	Onset of dyspnea on exertion
1 month before admission	Worsening dyspnea, new chest and abdominal pain, weight gain ∼9 kg
Day 1 (admission)	Physical examination, laboratory workup, CT chest: PE excluded, right atrial mass identified
Day 2	TTE and TEE: intracardiac mass confirmed
Day 3	Cardiac MRI: IVC tumor with right atrial extension confirmed
Day 5	Staging CT: high-grade suspicion of vascular tumor with advanced transthepatic IVC infiltration and tumor thrombosis extending into the right atrium
Day 6	Brain MRI: no cerebral metastases
Day 1–11	Decompensation therapy with furosemide initiated and continued throughout workup
Day 11	CT-guided transhepatic biopsy: low-grade spindle cell leiomyosarcoma G2 confirmed
After diagnosis	Anticoagulation initiated
Cycle 1–2	Neoadjuvant chemotherapy (doxorubicin/ifosfamide), well tolerated
Planned	Staging CT and TTE before cycle 4; surgical resection after 3–6 cycles

## 3 Discussion

Leiomyosarcoma of the inferior vena cava with extension into the right atrium is a rare and potentially aggressive malignancy, with fewer than 450 cases reported in literature (4). Clinical presentation depends on tumor location and extent of venous obstruction. In our case, progressive obstruction of the IVC resulted in clinical signs that mimic a right-sided heart failure. Although, intracardiac extension is uncommon, involvement of the suprahepatic IVC and the right atrium has been associated with significant worse prognosis and increased early mortality (5).

Our case highlights the central role of multimodality imaging for accurate differentiation between primary cardiac tumors and secondary intravascular malignancies. Due to its widespread availability, transthoracic echocardiography (TTE), remains the first line imaging modality for suspected intracardiac masses (6). In the presented case, TTE and TEE identified a vascularized mass within the right atrium. Despite providing important information on size, mobility and hemodynamic impact, echocardiographic examinations are unable to reliably determine the tumor origin due to its limitations in tissue characterization and in visualizing the full extent of IVC involvement, as well as adjacent extracardiac structures (6).

An important diagnostic challenge illustrated by this case, is the potential for malignant tumors to be misinterpreted as benign atrial myxomas. The initial CT scan, acquired in the pulmonary arterial phase as part of the evaluation for suspected pulmonary embolism, suggested an atrial myxoma, likely due to suboptimal contrast timing and limited differentiation between intracardiac and intravascular structures. Notably, leiomyosarcomas of the IVC typically appear on contrast-enhanced CT as heterogeneous masses with peripheral enhancement and central necrosis, sometimes accompanied by the characteristic “imperceptible IVC” sign (7). However, these features may not be reliably appreciated in pulmonary arterial phase imaging. This outlines a key limitation of CT in this setting and reinforces the need for complementary imaging modalities. CMR provided crucial information on both tissue characterization and tumor origin. Beyond its superior soft tissue contrast, CMR enables differentiation between benign and malignant masses based on morphology, signal characteristics, and contrast enhancement patterns (8). In this patient, the heterogeneous enhancement on late-gadolinium enhancement sequences, hyperintensity in T2-weighted images and isointensity in T1-weighted images strongly suggested malignancy. Beyond signal characterization, parametric mapping can provide quantitative tissue information in right atrial masses and may aid preoperative differential diagnosis (9). Most importantly, CMR clearly demonstrated continuity between the right atrial mass and the IVC, thereby establishing the diagnosis of an intravascular tumor rather than a primary cardiac neoplasm. These findings are consistent with previous reports demonstrating the diagnostic and prognostic value of CMR in patients with suspected cardiac tumors. In a large multicenter study, CMR showed high diagnostic accuracy and provided prognostic value beyond clinical risk factors (10).

Typical imaging characteristics of atrial myxomas on CMR further support this distinction. They are usually pedunculated and mobile masses attached to the interatrial septum, most commonly at the fossa ovalis. As a main differential diagnosis, myxomas often appear hyperintense on T2-weighted CMR images due to their high water content and show intermediate signal intensity on T1-weighted images. Contrast enhancement in myxomas is variable but frequently heterogeneous, reflecting their mixed composition of myxoid, cystic, and occasionally hemorrhagic components. Importantly, myxomas generally lack invasive growth and only rarely arise from the inferior vena cava, so continuity with extracardiac structures is uncommon and helps distinguish them from malignant tumors such as leiomyosarcoma (11).

In our patient, the site of origin settled the diagnosis. The mass did not arise from the interatrial septum but grew within the IVC and extended along the vessel into the right atrium, with CMR showing the two to be continuous. Its growth was broad-based and intraluminal rather than pedunculated, it was barely mobile, and it had no connection to the tricuspid valve. The IVC was dilated to 3 cm and largely obstructed. Myxomas differ in this respect. About 90% arise from the fossa ovalis by a narrow pedicle, and they may prolapse through the atrioventricular valve in diastole. Origin from the IVC is described but rare, and myxomas usually stay within the cardiac cavity (11). Growth along the vessel over the length seen here, with the caval dilatation and obstruction it caused, would be unusual for a myxoma. Where a right atrial mass extends into the IVC, lacks a pedicle, and moves little, an intravascular malignancy should be considered even if initial imaging suggests a benign lesion. Accurate differentiation between benign tumors such as atrial myxoma and leiomyosarcoma is of critical clinical importance, as it directly influences management and prognosis. Whereas atrial myxomas are typically treated with surgical resection alone and have good outcomes with low recurrence rates, leiomyosarcomas require a multimodal oncologic approach and are associated with a substantially worse prognosis (12, 13).

When considering follow-up imaging, it is helpful to distinguish between cardio-oncological monitoring and oncologic tumor staging. The 2022 guidelines of the European Society of Cardiology mainly focus on the assessment and surveillance of cardiovascular side effects related to cancer therapy, including recommendations for baseline evaluation and follow-up of cardiac function. They do not provide specific recommendations for the management or imaging follow-up of primary cardiac tumors or rare intravascular malignancies such as leiomyosarcoma (14). According to current guidelines from the European Society for Medical Oncology (ESMO) of 2021, imaging plays a central role in both initial staging and treatment response assessment in patients with soft tissue sarcomas. Contrast-enhanced CT is recommended for the detection of metastatic disease, particularly in the lungs, while MRI is preferred for local tumor evaluation (15). However, the guidelines do not define fixed imaging intervals for response assessment. Instead, tumor response is typically evaluated using cross-sectional imaging based on standardized criteria such as the Response Evaluation Criteria in Solid Tumors (RECIST) (16). During treatment, response assessment is commonly performed after a few cycles of systemic therapy using cross-sectional imaging, depending on the clinical context and treatment regimen. As outlined in the ESMO guidelines, patients at high risk may receive anthracycline–ifosfamide–based chemotherapy for at least three cycles. Against this background, response assessment including CT for systemic staging and TTE after three cycles in our patient reflects a clinically meaningful and commonly applied time point in sarcoma management (15).

A limitation of this report is the short follow-up. As the patient was still receiving neoadjuvant chemotherapy at the time of writing, surgical resection had not yet been performed, and long-term oncological and surgical outcomes are therefore not available and cannot be reported.

## 4 Conclusion

Intravascular leiomyosarcoma of the inferior vena cava with extension into the right atrium is a rare but important differential diagnosis of right atrial masses. This case illustrates that right atrial masses require thorough multimodality imaging evaluation. Recognition of tumor continuity with the IVC, as demonstrated by CMR, is a key feature distinguishing intravascular leiomyosarcoma from benign atrial myxoma. CMR should be regarded as an essential diagnostic step in patients with right atrial masses of uncertain origin, given its superior soft tissue characterization and ability to define extracardiac tumor extent. Early diagnosis and a multidisciplinary treatment strategy are important for optimal patient management.

## 5 Patient perspective

The patient reported that the diagnostic process, though lengthy, gave her confidence that the underlying cause of her symptoms was thoroughly investigated. She described the progressive dyspnea and abdominal swelling as increasingly distressing in the weeks prior to admission, and expressed relief when a definitive diagnosis was established. She tolerated the first two cycles of chemotherapy with manageable side effects and noted gradual improvement in her symptoms under diuretic therapy. She consented to the publication of this case in the hope that it may help clinicians recognize similar presentations earlier and spare other patients a diagnostic delay.

## Data Availability

The raw data supporting the conclusions of this article will be made available by the authors, without undue reservation.
